# Nanocrystal Formulation to Enhance Oral Absorption of Silybin: Preparation, In Vitro Evaluations, and Pharmacokinetic Evaluations in Rats and Healthy Human Subjects

**DOI:** 10.3390/pharmaceutics16081033

**Published:** 2024-08-02

**Authors:** SeungRee Seo, Gwan-Young Kim, Min-Hwan Kim, Kyung Won Lee, Min-Jae Kim, Mansingh Chaudhary, Khadka Bikram, Taeheon Kim, Seungmok Choi, Heejin Yang, Joo Won Park, Dae-Duk Kim, Ki-Taek Kim

**Affiliations:** 1 Life Science Research Institute, Daewoong Pharmaceuticals, Yongin-si 17028, Republic of Korea; 2220534@daewoong.co.kr (S.S.); pharmrich@daewoong.co.kr (G.-Y.K.); 2210471@daewoong.co.kr (M.-H.K.); thkim150@daewoong.co.kr (T.K.); smchoi344@daewoong.co.kr (S.C.); 2220176@daewoong.co.kr (H.Y.); 2 Bio-Synectics, Inc., Seoul 08826, Republic of Korea; c7512999@bio-synectics.com (K.W.L.); jenna@bio-synectics.com (J.W.P.); 3 College of Pharmacy and Research Institute of Pharmaceutical Sciences, Seoul National University, Seoul 08826, Republic of Korea; alswo7725@snu.ac.kr; 4 Department of Biomedicine, Health & Life Convergence Sciences (BK21 Four) and Biomedical and Healthcare Research Institute, Mokpo National University, Jeonnam 58554, Republic of Korea; mansingh9607@gmail.com (M.C.); khadkabikram180@gmail.com (K.B.); 5 Natural Products Research Institute, Seoul National University, Seoul 08826, Republic of Korea; 6 College of Pharmacy and Natural Medicine Research Institute, Mokpo National University, Jeonnam 58554, Republic of Korea

**Keywords:** milk thistle, silybin, nanocrystal, long-term stability, oral bioavailability, human pharmacokinetic

## Abstract

Despite the various therapeutic benefits and high tolerance of orally administered silybin, poor water-solubility can be the main restrictive physicochemical feature, which results in low oral bioavailability in the absorption. A milk thistle nanocrystal formulation (HM40) was prepared using a modified wet-milling method. Comprehensive characterization was performed to determine the physical morphology, crystallinity, and physicochemical properties. The long-term stability was evaluated over 24 months. In vitro silybin release was assessed at pH 1.2 for 2 h, followed by pH 6.8 for 4 h. Finally, in vivo pharmacokinetic studies were conducted in rats and healthy human volunteers. HM40 exhibited a nanocrystal structure maintaining crystallinity and enhanced the solubility and dissolution of silybin compared to that of the raw material. The stability over 24 months revealed consistent surface morphology, particle size, silybin content, and solubility. In vitro release profiles indicated a significant increase in the silybin release from HM40. In vivo pharmacokinetic studies demonstrated that HM40 showed 2.61- and 1.51-fold higher oral bioavailability in rats and humans, respectively, than that of the reference capsule. HM40 formulation presents a stable and promising approach for the oral delivery of poorly water-soluble silybin, with the potential for use in pharmaceutical formulations containing milk thistle.

## 1. Introduction

*Silybum marianum*, also known as milk thistle, has traditionally been used to treat and prevent liver disorders [1,2]. Milk thistle extract is composed of a mixture of flavonolignans, commonly known as silymarin, and is one of the most popular herbal supplements [1,2,3]. The main active component of silymarin is silybin, which has antioxidant and anti-cancer activities in the liver [3,4]. The hepato-protective effects of silybin are mediated by an increase in endogenous anti-oxidant molecules (e.g., glutathione and superoxide dismutase), a reduction in reactive oxygen species, and inhibition of necroinflammation in the liver [4,5,6]. Based on these effects, commercial products (i.e., Legalon^®^ and Enerthistle^®^) as a hard capsule or tablet formulation containing 70–140 mg silymarin have been launched in the market and are orally administered twice a day [7]. Several studies have shown that high doses of silybin are well-tolerated in animals and humans. The LD_50_ (50% lethal dose) of oral silybin is 10,000 mg/kg in rats [8]. In healthy humans, oral administration of 400 mg silymarin once a day for 63 days exhibited no significant toxicity [9]. Moreover, oral silymarin was also well-tolerated by the patients with chronic hepatitis C even at a high dose of 700 mg thrice daily for 24 weeks [10]. Silybin exhibits low drug–drug interactions and does not have major effects on cytochrome P-450 at its therapeutic dose range in humans [11,12]. However, it is classified as a class II compound under the Biopharmaceutics Classification System (BCS). Thus, despite the various therapeutic benefits and high tolerance of orally administered silybin, poor water-solubility (≤50 μg/mL) can be the main restrictive physicochemical feature, which results in low oral bioavailability (23–47%) and large variability in the absorption [13,14,15].

Therefore, diverse formulation strategies to increase the solubility of silybin have been widely investigated. Pharmaceutical approaches reported in the literature include oil/lipid-based formulations (e.g., emulsions, self-emulsifying drug delivery systems, liposomes, and solid lipid nanoparticles), polymer-based nanoparticles, cyclodextrin inclusion complexes, and solid dispersions [1,7,13,16,17,18,19]. Although these approaches may increase the oral absorption of silybin, there are significant risks associated with long-term stability. Formulations in which an active pharmaceutical ingredient (API) is dispersed as an amorphous state within hydrophilic polymer matrices may experience a “re-crystallization” phenomenon wherein the amorphous API returns to the crystalline state over time, leading to decreased drug solubility and release [20]. Furthermore, in cases where the final dosage form is a liquid, irreversible destruction may occur because of the aggregation and sedimentation of dispersed particles during storage [21]. Additionally, there are limitations in terms of industrial applicability owing to challenges in mass production and reproducibility [21,22].

Nanocrystals have emerged as potential nano-drug delivery systems for poorly water-soluble drugs since the 20th century [23]. They are pure drug particles dispersed in an aqueous medium with particle sizes in the range of 1–1000 nm [24]. In contrast to other nano formulations, nanocrystals do not require a matrix or carrier, which can minimize carrier-related toxicity [25]. In addition, high drug content in the nanocrystal formulations is beneficial for mass production [26]. However, during the preparation and storage processes, aggregation and sedimentation can occur due to the high surface energy caused by size reduction, resulting in physical instability [27]. To solve these problems, the addition of stabilizers such as biocompatible surfactants and polymers (e.g., polysaccharides) is required to inhibit nucleation and crystal growth, and prevent collisions and aggregation between particles [28,29]. Nanocrystal preparation methods can be categorized into bottom-up (e.g., anti-solvent precipitation and supercritical fluid (SCF) methods) and top-down (e.g., high-pressure homogenization and wet-milling) methods [23,30]. Bottom-up methods involve dissolving the API in a solvent (usually an organic solvent) or a supercritical fluid (SCF) (e.g., CO_2_), and then precipitating the API by adding an anti-solvent or vaporizing the fluid through a nozzle spray [30]. Compared with top-down methods, these methods provide simplicity in operation and better control of particle properties but are difficult to scale up owing to poor reproducibility [31]. Additionally, the use of organic solvents in the preparation process leads to problems with solvent residues [23]. Based on these limitations, the top-down methods have usually been selected for producing nanocrystals in the market (e.g., Emend^®^, aprepitant nanocrystal; Tricor^®^, fenofibrate nanocrystal; and Ritalin^®^ LA, methylphenidate nanocrystal) [32]. Nanocrystals obtained by the high-pressure homogenization method exhibit a small particle size with a narrow distribution [33]. However, high pressure may lead to changes in the crystal structure and increase the content of amorphous states, thus resulting in physical instability of the nanocrystals [34]. In the wet-milling method, a mixed paste of the crude drug and stabilizers is put into a chamber filled with grinding media (e.g., zirconia, glass, or agate coated with polystyrene resin) [30]. High-speed rotation of the chamber facilitates interactions among the paste, grinding media, and container walls, generating a continuous shear force that provides the requisite energy for micronizing the drug particles [35]. 

Several studies reported nanocrystal formulations of silybin (or silymarin) to enhance its solubility by adapting anti-solvent precipitation, SCF, high-pressure homogenization, and wet-milling methods [14,36,37,38,39,40,41]. Wang et al. applied a wet-milling method with zirconia beads as grinding media to prepare silybin nanocrystals, reporting a significant increase in oral absorption [41]. However, the hard beads of the grinding media, made of inorganic substances such as zirconia, glass, and alumina, could possibly contaminate the nanocrystals [36], yet the wet milling method without grinding media has not been reported. Thus, the objective of this study was to develop a nanocrystal formulation of silybin by a modified wet-milling method without conventional grinding media, using a self-made roller kneader and edible polysaccharides as stabilizers. The formation of nanocrystals was confirmed by observing their physical morphology, crystallinity, and physicochemical properties including particle size. In addition to the long-term stability of the nanocrystal formulations for 24 months, an in vitro silybin release study and in vivo pharmacokinetic studies in rats and healthy human volunteers were performed.

## 2. Materials and Methods

### 2.1. Materials

Milk thistle raw material containing 38.3 ± 0.4% (*w*/*w*) silybin was purchased from Indena S.p.A. (Settala, Italy). Xanthan gum and gum ghatti were obtained from Cargill, Inc. (Baupte, France) and APD Foods India Pvt., Ltd. (Mumbai, India), respectively. Enerthistle^®^ capsules were gifted from Daewoong Pharmaceutical Co., Ltd. (Seoul, Republic of Korea). The columns, reagents, and solvents for high-performance liquid chromatography (HPLC) and HPLC-tandem mass spectroscopy (MS/MS) analyses were obtained from Merck and Thermo Fisher Scientific (Waltham, MA, USA).

### 2.2. Preparation of Nanocrystal Formulation

**Figure 1:**
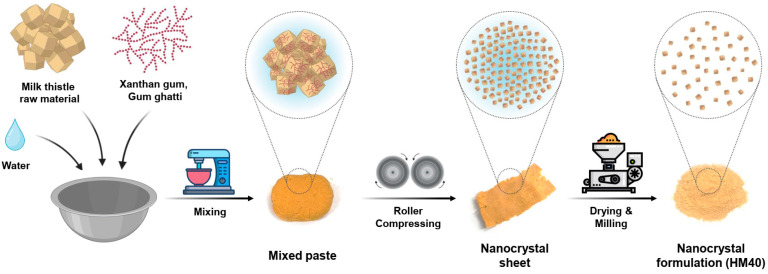
Schematic illustration of the HM40 preparation process.

The milk thistle nanocrystal formulation (HydroMilkthistle^TM^, HM40) was prepared using a modified wet-milling method (Figure 1) [42]. Briefly, milk thistle raw material, xanthan gum, and gum ghatti were mixed in a 1:0.1:0.4 (*w*/*w*) ratio. A small amount of deionized (DI) water was added, and the resulting paste was prepared using a vertical mixer (VM-40; Hunwoo, Seoul, Republic of Korea). Next, the paste was pressed using a self-made roller kneader (Bio-Synectics, Seoul, Republic of Korea), and the compressed sheet was dried overnight in a vacuum-drying oven (MG-VAV270S; MG Industry, Gunpo, Korea) at 35 °C. The dried sheets were pulverized using a pin mill (PJ-S10; PoongJin Food Machine, Pyeongtaek, Republic of Korea) to produce the HM40 powder. 

### 2.3. Physical Morphology and Physicochemical Characterization of HM40

#### 2.3.1. Measurements of Physical Morphology

The surface morphologies of the milk thistle raw material and HM40 powder were characterized using field-emission scanning electron microscopy (FE-SEM, 7610F, JEOL, Tokyo, Japan) at an accelerating voltage of 5.0 kV. Before observation, the samples were sputtered onto a carbon tape and coated with a thin layer of platinum under vacuum. The morphology of the aqueous dispersion of milk thistle raw material and HM40 was also observed using FE-SEM. Briefly, the aqueous dispersions (2.0 mg/mL as silybin) were filtrated using a syringe filter (pore size 0.20 μm, Minisart; Satorius, Goettingen, Germany), and the remained samples on the filter were dried using a dry oven, followed by coating with platinum under vacuum. 

The morphology of the HM40 aqueous dispersion was observed by a transmission electron microscopy (TEM; LIBRA 120, Carl Zeiss, Oberkochen, Germany) at 80 kV. A drop of the aqueous dispersion (2.0 mg/mL as silybin) was loaded onto a carbon-coated Cu grid and negatively stained with 2% uranyl acetate. The mean particle size and size distribution of HM40 were measured using a dynamic light-scattering (DLS) analyzer (SZ-100; HORIBA, Osaka, Japan), where the HM40 aqueous dispersion (2.0 mg/mL as silybin) was transferred to a 3 mL quartz cuvette. All the measurements were performed at 25 °C.

The crystallinity of the milk thistle raw material, HM40, and other excipients was assessed using a powder X-ray diffractometry (pXRD; D8 ADVANCE with DAVINCI, BRUKER, Berlin, German) equipped with Cu Kα1 radiation at λ = 1.5418 Å. Acceleration voltage and current of 40 kV and 40 mA, respectively, were used. Samples were scanned in a 2-theta (2θ) range of 3–45° with a step angle of 0.02°. Thermal analysis was performed using differential scanning calorimetry (DSC; Q1000, TA Instruments, New Castle, DE, USA) to evaluate the thermal transition properties of the milk thistle raw material, HM40, and other excipients. Accurately weighed samples were sealed in aluminum pans and scanned from 30 to 190 °C at a heating rate of 10 °C/min. 

#### 2.3.2. Characterization of Physicochemical Properties

To measure the silybin content (%) in HM40, as an indicator, 100 mg of the powder formulation was disrupted with 100 mL of methanol via sonication and adequately diluted with methanol. The concentrations of silybin in the samples were determined using an HPLC system (Waters Alliance 2695; Waters Co., Milford, MA, USA), equipped with a UV/PDA detector (Waters 996; Waters Co., Milford, MA, USA) at a wavelength of 288 nm and a C18/ODS column (250 × 4.6 mm, 5 μm, 100 Å; ES Industries, West Berlin, NJ, USA) [43]. The mobile phase consisted of solvent A (a mixture of water and methanol [80:20, *v*/*v*] with 0.5% phosphoric acid) and solvent B (a mixture of water and methanol [20:80, *v*/*v*] with 0.5% phosphoric acid) at a flow rate of 1.0 mL/min. The initial composition of the elution gradient was maintained at 85% solvent A for 5 min, linearly changed to 55% (5–25 min) and maintained for 30 min (25–55 min). The gradient elution was then returned to the initial composition within 1 min (55–56 min) and maintained for 14 min (56–70 min) to achieve the column equilibrium. Aliquots (20 μL) of the samples and standard solutions (1, 2, 5, 10, 20, 50, and 100 μg/mL) were injected into the column. The retention time of silybin was 29.8 min. The lower limit of quantification (LLOQ) of analyte was 0.585 μg/mL. The linearity of calibration curve over the concentration range of 0.585–58.5 μg/mL as silybin was over 0.999.

After determining the silybin concentration in the samples, the following equation was used to calculate the silybin content (%) in the HM40 powder:

(1) $$Silybin\;content\;(\%)=\frac{A}{A_{0}}\times 100,$$

where A is the amount of silybin measured by HPLC analysis (mg) and A_0_ is the amount of HM40 powder (100 mg).

The saturated aqueous solubility of silybin achieved from milk thistle raw material and HM40 was measured by adding excess amounts (10 mg) of each sample to DI water (1 mL). The mixtures were allowed to reach an equilibrium state in a vortex shaker (Vortex-Genie 2; Scientific Industries, Inc., Bohemia, NY, USA) at 25 °C for 1 day. The equilibrated samples were centrifuged at 12,000× *g* for 7 min, and the supernatants were centrifuged again at 12,000× *g* for 7 min to remove the undissolved silybin. The amount of dissolved silybin from the double-centrifuged samples was quantified using HPLC.

### 2.4. Long-Term Stability of HM40

The long-term stability of HM40 was assessed by determining changes in the mean particle size, silybin content, surface morphology, and silybin solubility. HM40 powder was stored in a stability chamber at 25 °C and 60% relative humidity (RH) condition. At each time point (0, 1, 3, 6, and 24 months), the mean particle size and size distribution of the samples after dispersing in DI water were measured using a DLS analyzer, while the silybin content of the samples was determined by HPLC. After 24 months of storage, the surface morphologies of the HM40 powder and its aqueous dispersion were observed by FE-SEM and compared with those of the initial state of HM40 (0 month). The saturated aqueous solubility of silybin achieved from HM40 after 24 months of storage was measured using HPLC and compared with that achieved from the initial milk thistle raw material and HM40 (0 month).

### 2.5. In Vitro Release Study

The in vitro release of silybin from milk thistle raw material and HM40 was measured using a United States Pharmacopeia (USP) type II dissolution apparatus (Hanson Vision Elite 8 Dissolution tester; Hanson Research Co., Chatsworth, CA, USA) with a paddle speed at 75 rpm and temperature of 37.0 ± 0.2 °C. The pH of the medium was adjusted from pH 1.2 to pH 6.8 at 2 h to investigate the release in the gastrointestinal (GI) tract, as reported in the literature with slight modifications [44,45]. Briefly, each formulation equivalent to 75 mg silybin was dispersed in grapeseed oil and encapsulated in a soft gelatin capsule. They were placed with a sinker in 750 mL of pH 1.2 medium (0.1 N HCl solution) containing 3% (*w*/*v*) tween 80, and the release study was conducted for 2 h. Then, 250 mL of 0.2 M tribasic sodium phosphate (Na3PO4) with 3% (*w*/*v*) tween 80 was added to adjust the pH of the medium to 6.8, and the release study lasted for 4 h. Aliquots (1 mL) of the release media were collected from the dissolution vessels at predetermined time intervals (0, 1, 2, 3, 4, and 6 h), and an equal volume of fresh medium was replenished. The samples were immediately filtered through a 0.2 μm syringe filter, and properly diluted with the mixture of media and methanol (1:1, *v*/*v*) for HPLC analysis.

### 2.6. In Vivo Pharmacokinetic Study in Rats

In vivo pharmacokinetics of silybin were investigated in rats after oral administration of milk thistle raw material or HM40 in Sprague–Dawley (SD) rats at a dose of 200 mg/kg as silybin. Male SD rats (6–7-week-old, 200–250 g) were obtained from Koatech (Pyeongtaek, Republic of Korea), and were housed at 22 ± 3 °C and 55 ± 15% RH. They were maintained under a 12 h light/dark cycle and provided with free access to food and water. The animal experimental protocol was approved by the Institutional Animal Care and Use Regulations of the Institutional Animal Care and Use Committee (IACUC) of Daewoong Pharmaceutical Institute (IACUC-23-012). 

The rats were fasted overnight before oral administration, and each powder was dispersed in an aliquot (1.0 mL) of DI water immediately before the administration. Blood samples (200 μL) were collected from the jugular vein at predetermined time points (0, 0.25, 0.5, 1, 2, 4, and 6 h) after oral administration. The collected samples were centrifuged at 16,000× *g* for 5 min at 4 °C, and the plasma was stored at −70 °C until HPLC-MS/MS analysis.

The plasma samples (50 μL each) were vortex-mixed with 200 μL of acetonitrile (ACN) containing 1.0 ng/mL of niclosamide as an internal standard (IS) for 5 min and centrifuged at 16,000× *g* for 5 min. Aliquots (2 μL) of the supernatant were injected into an HPLC–MS/MS system equipped with an HPLC system (Exion; AB Sciex, Framingham, MA, USA) and a Triple Quad 5500 mass spectrometer (API5500 TQ; AB Sciex). The chromatographic separation of silybin and IS was performed on Kinetex^®^ C18 column (2.1 × 50 mm, 5 μm, 100 Å; Phenomenex) at 40 °C. The mobile phase consisted of solvent A (water containing 0.1% formic acid) and solvent B (acetonitrile (ACN) containing 0.1% formic acid) at a flow rate of 0.4 mL/min. The initial composition of gradient elution was maintained at 10% solvent B for 0.2 min, changed linearly to 70% (0.2–1.2 min) and maintained for 0.8 min (1.2–2.0 min). The gradient elution was then returned to the initial composition within 0.2 min (2.0–2.2 min) and kept 0.8 min (2.2–3.0 min) to achieve the column equilibrium. An electrospray ionization interface was used as the ion source in negative-ion multiple-reaction monitoring mode. The *m*/*z* values of the precursor to product ion for silybin and IS were 481.0 → 125.0 and 325.0 → 170.9, respectively. The retention time of silybin and IS was 1.5 min and 2.1 min, respectively. The LLOQ of analyte was 3.0 ng/mL. The linearity of plasma calibration curve over the concentration range of 3.0–3000 ng/mL as silybin was 0.991. 

### 2.7. Pharmacokinetic Study in Health Human Subjects

#### 2.7.1. Human Subjects

The pharmacokinetic study was conducted at Raptim Research Pvt., Ltd. (Navi Mumbai, India) in healthy volunteers (18–45-year-old; one female and 13 males). All participants were determined to be in good health based on their medical history, and physical and hematological examinations. Participants were excluded if they had hypersensitivity to milk thistle or its formulations, took any prescribed medicine or over-the-counter (OTC) products (e.g., herbal medicines and vitamin supplements) within 30 days before the first check-in and throughout the study, or had difficulty in swallowing tablets or capsules. All subjects signed a written informed consent form after the purpose, methods, and adverse drug reactions of the study were explained to them. The participants were monitored by hospital staff during the study period using interviews, vital sign measurements, adverse event recordings, and physical examinations.

#### 2.7.2. Study Design

This study was performed under fasting conditions using a randomized, single-dose, two-period crossover design. All subjects (*n* = 14) received a single dose (75 mg as silybin) of reference capsule (Enerthistle^®^) or HM40 capsule (same as the in vitro release study), with 7 days of washout period between treatments. The study protocol was approved by an Independent Human Care Ethics Committee and followed the principles of the Declaration of Helsinki (protocol code: PR/BE/23/242).

The subjects were hospitalized one day before the study, and then exercise, meals, smoking, and consumption of grapefruit juice were restricted from 10 h before the beginning of the trial to the end of blood collection. During the trial, consumption of food and drinks, except water, was controlled. The subjects fasted for 10 h before and 4 h after oral administration of the capsules to exclude the effects of diet. The reference capsule or HM40 capsule was orally administered with 240 ± 2 mL of water at 8 am. Meals were provided at 4 and 8 h after dosing. After Period-1 blood collection, all subjects returned home and were advised to avoid excessive alcohol and tobacco use, drug intake, and drinking grapefruit juice and to prohibit excessive intake of foods containing xanthine derivatives (e.g., chocolates, coffee, and tea). After 7 days for washout period, all subjects were hospitalized one day before the study, and the capsules were administered in the same manner as in Period-1.

Blood samples were collected at predetermined time points (0, 0.167, 0.334, 0.5, 0.75, 1, 1.25, 1.5, 1.75, 2, 2.334, 2.667, 3, 3.5, 4, 6, and 8 h). Aliquots (5 mL) of blood were collected in a vacutainer containing sodium heparin and 2 mL of heparinized normal saline was injected into the catheter to prevent blood clotting. Blood samples were centrifuged at 16,000× *g* for 10 min at 5 ± 3 °C, and the plasma was stored at −70 °C until HPLC-MS/MS analysis.

#### 2.7.3. Analysis of Silybin Concentration in Human Plasma

The plasma concentration of silybin in humans was analyzed using an HPLC–MS/MS system equipped with an HPLC system (Exion; AB Sciex) and a triple quad 4500 mass spectrometer (API4500; AB Sciex). The chromatographic separation of silybin and silybin-d3 as an IS was performed on Hypurity^TM^ C18 column (4.6 × 50 mm, 5 μm, Thermo Scientific, Waltham, MA, USA) at 40 °C. The mobile phase consisted of 2 mM ammonium acetate containing 0.2% formic acid in DDW and acetonitrile (20:80, *v*/*v*), at a flow rate of 0.4 mL/min. The *m*/*z* values of the precursor to product ion for silybin and IS were 481.0 → 125.0 and 484.1 → 125.0, respectively. 

The plasma samples (200 μL each) were vortex-mixed with 25 μL of IS stock solution for 30 s. Then, 200 μL of 1 mM NaH_2_PO_4_·H_2_O solution was added and vortexed for 30 s. Subsequently, 3 mL of tert-butyl methyl ether solution was added, and the mixture was vortexed for 5 min. All samples were put into the Rotospin for 15 min, followed by centrifugation at 4000 rpm for 10 min at 4 °C. The supernatant (2 mL) was transferred to clean tubes and allowed to evaporate under a steam of nitrogen gas at 50 °C. The residue was reconstituted with 200 μL of mobile phase and vortexed for 30 s. Aliquots (5 μL) of the samples were injected into the column.

### 2.8. Pharmacokinetic Parameters and Statistical Analysis

Pharmacokinetic parameters were calculated using non-compartmental analysis (Phoenix WinNonlin, version 8.3.4.295; Certara, CA, USA): peak plasma concentration (C_max_), time to reach C_max_ (T_max_), total area under the plasma concentration–time curve from time zero to infinity (AUC_inf_), mean residence time (MRT), and relative bioavailability. All experiments were repeated at least three times, and the data are expressed as the mean ± standard deviation. SPSS statistics software (Version 21.0; IBM Corp, New York, NY, USA) was used to perform statistical analyses using Student’s *t*-test and one-way analysis of variance (ANOVA) with Tukey’s test for post-hoc comparisons. A *p* value of <0.05 was considered statistically significant.

## 3. Results and Discussion

### 3.1. Preparation and Characterization of HM40

A milk thistle nanocrystal formulation (HM40) was prepared by a modified wet-milling method using a self-made roller kneader without conventional grinding media. This process can prevent a possible contamination from conventional grinding media, including zirconia, glass, and polystyrene resin. During the roller-compression of the mixed paste using the roller kneader, the crude milk thistle raw material was compressed by the pressure of rotating heavy rollers, resulting in size reduction (mean particle size; 645 nm, polydispersity index [PDI]; 0.640) and nanocrystal formation (Supplementary Figure S1) [46,47]. Moreover, xanthan gum and gum ghatti are edible polysaccharides and non-toxic pharmaceutical excipients, and thus usually added as a stabilizer in various pharmaceutical formulations, including nanocomplex [48,49,50]. These polysaccharides are known to prevent the coalescence and/or aggregation of nano-sized particles by increasing the viscosity of liquid medium [48]. The pulverized final HM40 formulation was beige color powder containing 26.0 ± 0.1% of silybin (Figure 1 and Table 1), which was known as a major active pharmaceutical ingredient (API) of milk thistle and its analytic indicator. Since milk thistle raw material containing 38.3% silybin was mixed with excipients (xanthan gum and gum ghatti) at a 1:0.5 (*w*/*w*) ratio, the 26.0% silybin content in the HM40 formulation indicated that the loss of API during the preparation process was negligible.

**Figure 2:**
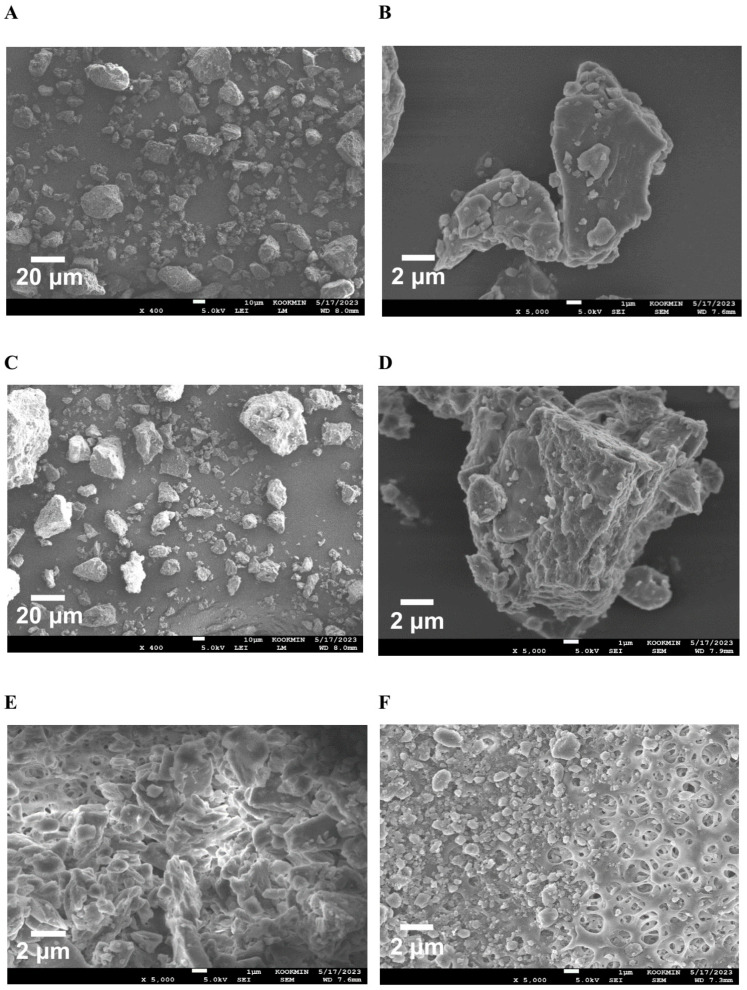
FE-SEM images of the powder form of (**A**,**B**) milk thistle raw material, and (**C**,**D**) HM40. FE-SEM images of (**E**) milk thistle raw material, and (**F**) HM40 particles after filtration (0.20 μm) of their aqueous dispersion.

**Figure 3:**
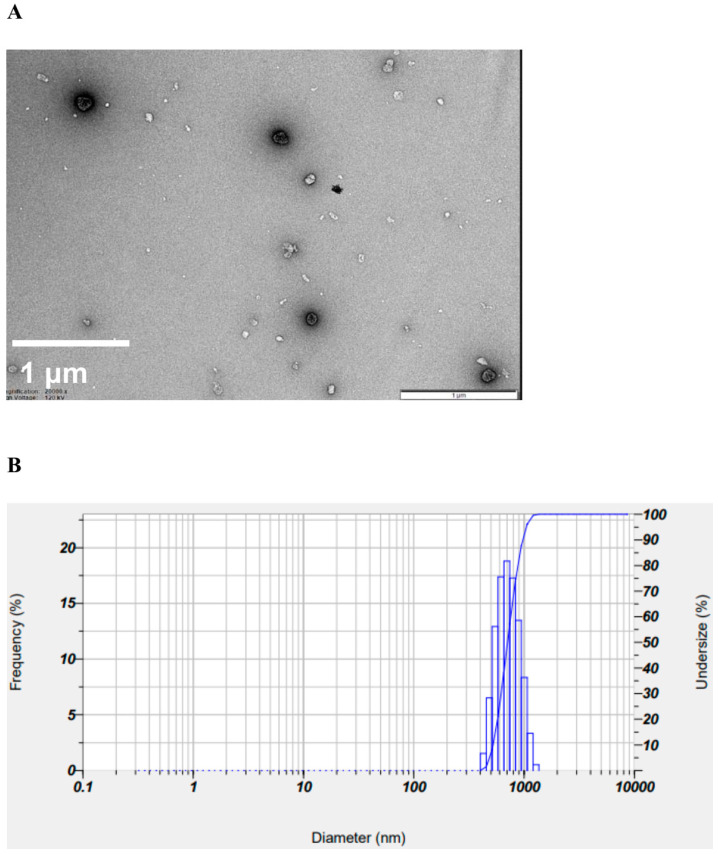
(**A**) TEM images, and (**B**) size distribution of HM40 aqueous dispersion.

To confirm the surface morphology of HM40, FE-SEM images of HM40 were compared with those of the milk thistle raw material (Figure 2). Milk thistle raw material exhibited a distinct angular crystalline structure (Figure 2A,B). A similar crystal structure was observed in the HM40 powder (Figure 2C,D), indicating that the prepared powder form of the nanocrystal formulation (HM40) did not alter the crystalline structure of the milk thistle raw material. It is interesting to observe that the powder obtained from the aqueous dispersion of the milk thistle raw material exhibited the formation of large, aggregated particles (Figure 2E), suggesting that the milk thistle raw material did not disperse properly in water. However, it is notable that the powder obtained after filtering and drying of the dispersed HM40 in DI water exhibited relatively spherical particles with a size of <1 μm (Figure 2F). These results indicate that the HM40 powder can be effectively dispersed in water with nano-sized particles. Thus, xanthan gum and gum ghatti could have served as dispersants and stabilizers, inhibiting aggregation of milk thistle when the HM40 formulation was dispersed in water [48,49,50]. In accordance with these results, TEM images of HM40 aqueous dispersion demonstrated the formation of spherical and nano-sized particles of less than 1 μm in diameter (Figure 3A). The particle size of HM40 aqueous dispersion measured by DLS analyzer exhibited 637 ± 43 nm and PDI of 0.464 ± 0.024 (Figure 3B, Table 1). The zeta potential of HM40 aqueous dispersion was −38.7 ± 3.5 mV, which could be attributed to xanthan gum that has negative-charged functional group [51]. Particles with a high zeta potential can contribute to the increased stability of their aqueous dispersion owing to repulsive forces between the particles [51,52].

**Figure 4:**
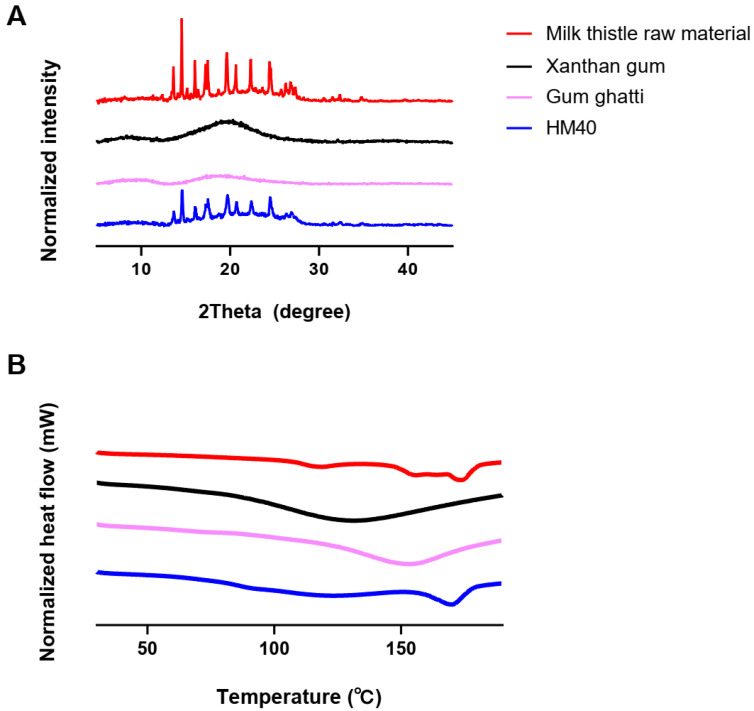
(**A**) XRD patterns, and (**B**) DSC thermograms of milk thistle raw material, excipients, and HM40.

pXRD and DSC analyses were performed to investigate the changes in the crystallinity and endothermic peaks of milk thistle in the HM40 powder, thereby confirming the encapsulation or incorporation of milk thistle into the excipients (xanthan gum and gum ghatti). As shown in Figure 4A, the milk thistle raw material exhibited sharp peaks in a 2θ range of 13° to 27°, which is consistent with a typical crystalline structure of milk thistle in the literature [53]. Moreover, the thermal curves for the milk thistle raw material showed a narrow endothermic peak at 170 °C, indicating characteristics of the crystalline forms (Figure 4B) [53]. Notably, the HM40 exhibited the sharp peaks in the same 2θ angles and the narrow endothermic peak at the same temperature as those of the milk thistle raw material (Figure 4A,B). This indicated that the prepared nanocrystal formulation was not a drug-encapsulated or drug-incorporated form and maintained the crystallinity of milk thistle, which was in accordance with the FE-SEM images of the milk thistle raw material and HM40 powder (Figure 2). Similarly, nanocrystal formulations maintaining the drug crystallinity have been reported in other literature [54,55]. Amorphization technologies such as solid dispersion and nano-encapsulation formulations, are commonly used to enhance the solubility of poorly water-soluble drugs [56,57]. However, the main drawback of these approaches is the possibility of amorphous drugs to undergo “re-crystallization” during storage, resulting in decreased solubility and dissolution rate, as well as lowered in vivo performance [58]. In contrast, the prepared nanocrystal formulation (HM40) did not affect the crystallinity of milk thistle, indicating the possibility of a significant improvement in physical stability during storage [54,55]. 

### 3.2. Long-Term Stability of HM40

The stability of a formulation is the extent to which the formulation stored under specified conditions maintains properties and characteristics similar to those in the initial state. The long-term stability of HM40 was evaluated at 25 °C and 60% RH for 24 months. As shown in Table 1, the silybin content ranged from 100 ± 1% to 103 ± 2% over 24 months, indicating that there was no significant change in the silybin content in the formulation during storage. In addition, the HM40 particles after dispersing in DI water at each time point showed a consistent particle size and PDI for 24 months, indicating that the overall long-term storage stability of the HM40 powder was maintained. 

**Figure 5:**
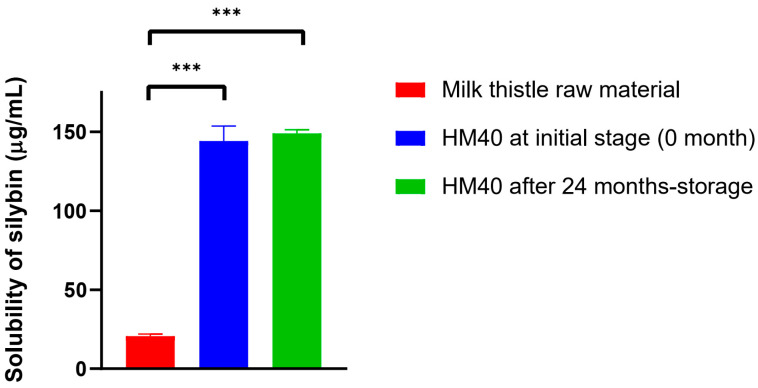
The saturated aqueous solubility of silybin achieved from milk thistle raw material and HM40, and that from HM40 after 24 months of storage at 25 °C and 60% RH condition (*** *p* < 0.001, compared to Milk thistle raw material).

The water-solubility of silybin from HM40 (144 ± 9 μg/mL) was significantly enhanced compared to that from milk thistle raw material (20.6 ± 1.3 μg/mL) (Figure 5). This can be attributed to the presence of nano-sized silybin particles in HM40 aqueous dispersion (Figure 2F and Figure 3), leading to an increase in the surface area [59]. Moreover, the saturated aqueous solubility of silybin achieved from HM40 after 24 months of storage (149 ± 2 μg/mL) was not significantly different from that of the initial HM40 (144 ± 9 μg/mL) (Figure 5). These results demonstrate that the storage of HM40 for 24 months did not affect long-term solubility, whereas the long-term storage stability of various formulations containing API in an amorphous state was affected by re-crystallization during storage. This could be attributed to the high stability of the API in the HM40 formulation in the form of nano-sized crystallinity without aggregation [54,55].

**Figure 6:**
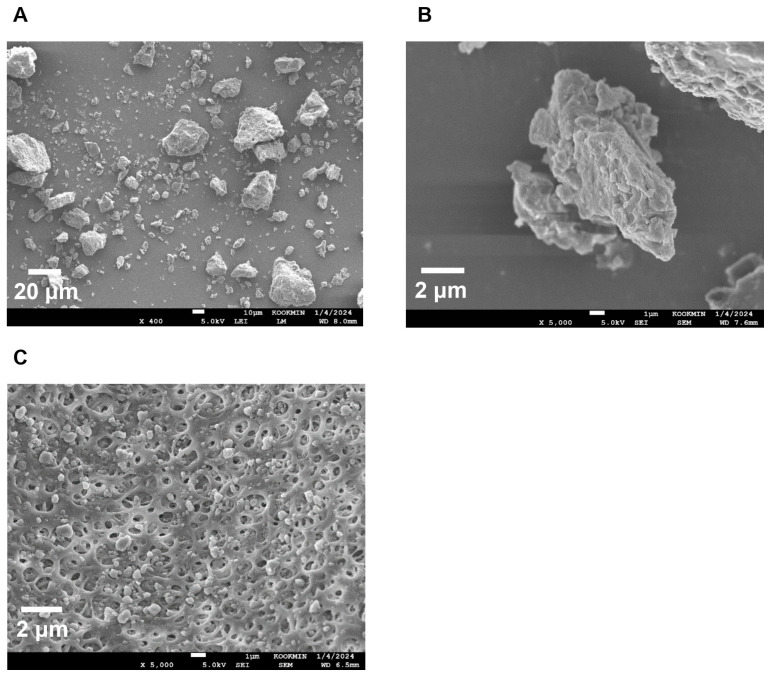
FE-SEM images of (**A**,**B**) powder form of HM40 stored for 24 months at 25 °C and 60% RH condition, and (**C**) HM40 particles after filtration (0.20 μm) of its aqueous dispersion.

The morphology of HM40 stored for 24 months was compared with that of the initial state using FE-SEM to elucidate the mechanism of maintenance of solubility over long-term storage. As shown in Figure 6A,B, the crystalline structure of the HM40 powder after 24 months of storage was consistent with that of the initial powder (Figure 2C,D). Moreover, HM40 particles in an aqueous dispersion of HM40 stored for 24 months (Figure 6C) maintained their sizes of <1 μm and exhibited no significant changes compared to the initial state (Figure 2F), suggesting maintenance of nano-size crystalline state in HM40 formulation.

### 3.3. In Vitro Release Study

**Figure 7:**
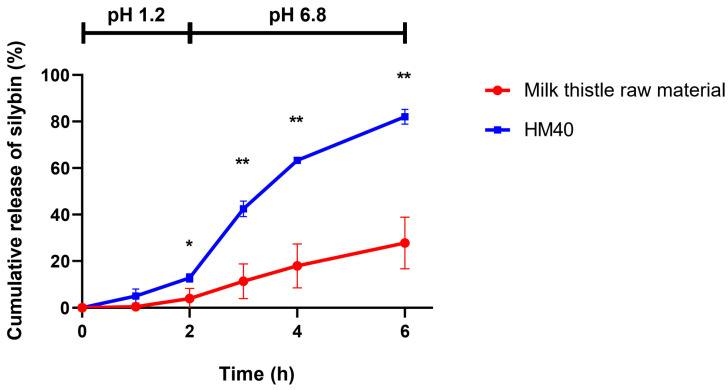
In vitro release of silybin from milk thistle raw material and HM40 in buffer-changed condition. The pH of the release medium was change from pH 1.2 to pH 6.8 after 2 h (* *p* < 0.05; ** *p* < 0.01, compared to milk thistle raw material).

Figure 7 shows the in vitro release profiles of silybin from milk thistle raw material and HM40 formulation, which were performed at two different pH conditions (pH 1.2 for 2 h, followed by pH 6.8 for 4 h) to simulate the physiological environment of the GI tract. In both release media, 3% (*w*/*v*) tween 80 was added to maintain the sink conditions. In pH 1.2 medium, the release of silybin from both milk thistle raw material and HM40 formulation was lower than that in pH 6.8 medium, probably because of the delayed disintegration of the soft capsules. Notably, however, the amount of silybin released from HM40 was significantly enhanced up to 82.1% of the initial amount at 6 h, compared to the milk thistle raw material with 27.8% release. Thus, it was expected that oral administration of the HM40 formulation would enhance the dissolution of silybin in the GI tract, which in turn would lead to an increase in the oral bioavailability of silybin.

The Noyes–Whitney equation describes the rate of dissolution of a solid material in media, as follows:

(2) $$\frac{dM}{dt}=\frac{D\cdot S}{h}C_{s}$$

where, $\frac{dM}{dt}$ is the dissolution rate, D is the diffusion coefficient, S is the surface area, h is the thickness of the diffusion layer, and C_S_ is the saturation concentration [60]. Based on this equation, enhanced release of silybin from the nanocrystal formulation, HM40, can be explained by the following mechanisms: (1) increase in the surface area by particle size reduction, (2) decrease in the thickness of the diffusion layer, and (3) increase in the silybin solubility by size reduction to the nano-sized crystal [60].

### 3.4. In Vivo Pharmacokinetic Study in Rats

**Figure 8:**
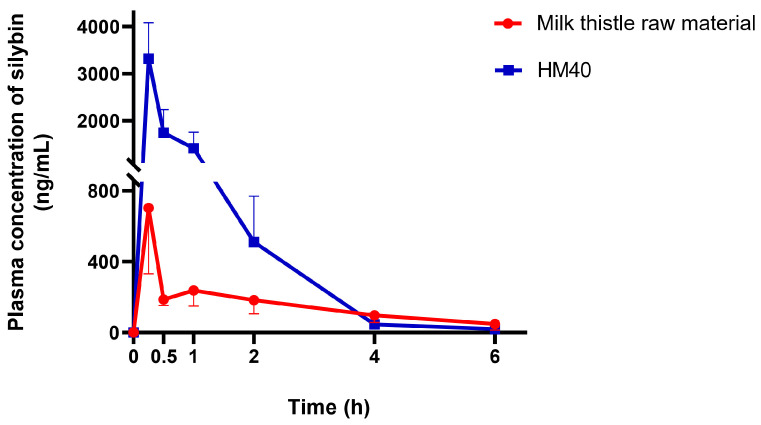
Plasma concentration–time profiles of silybin after oral administration of milk thistle raw material or HM40 at a dose of 200 mg/kg as silybin in rats (*n* = 5).

The plasma silybin concentration–time profiles after oral administration of milk thistle raw material or HM40 in an aqueous dispersion (equivalent to 200 mg/kg as silybin) are depicted in Figure 8. The mean pharmacokinetic parameters are presented in Table 2. The plasma concentration of silybin reached its maximum 0.25 h after the oral intake of milk thistle raw material and HM40, indicating rapid absorption through the GI membrane. It is interesting to note that oral administration of an aqueous dispersion of HM40 resulted in rapid absorption with a short T_max_ value, whereas the in vitro release of silybin from the soft gelatin capsule was delayed owing to the disintegration process (Figure 7). The values of C_max_ and AUC_inf_ after oral administration of HM40 were 4.72- and 2.61-fold higher, respectively, than those of the milk thistle raw material, resulting in 2.6-fold higher oral bioavailability. Enhanced oral bioavailability could be attributed to the increased solubility and dissolution of silybin after preparation of the nanocrystal formulation [61]. Additionally, nanocrystals could have enhanced their adhesion to the mucous layer of epithelial cells, thereby increasing silybin absorption through the GI tract [59]. Notably, the MRT after oral administration of HM40 was significantly lower than that of milk thistle raw material. Because silybin is poorly water-soluble, its dissolution from milk thistle raw material can be the rate-determining step of GI absorption, leading to increased MRT [62]. In contrast, HM40, which enhanced the solubility of silybin, exhibited improved dissolution rates, resulting in a significant reduction in MRT.

### 3.5. Pharmacokinetic Study in Healthy Human Subjects

**Figure 9:**
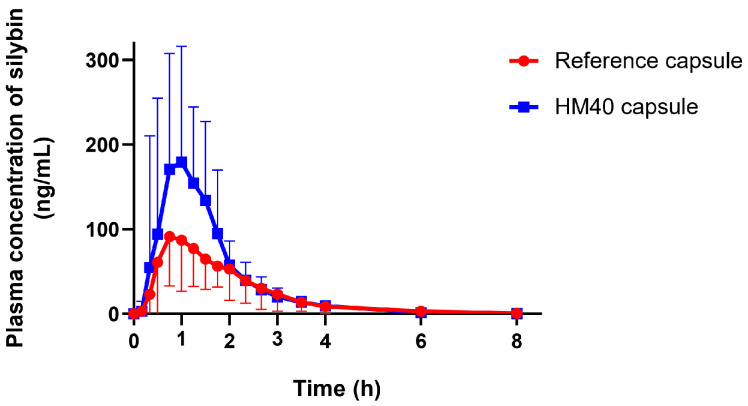
Plasma concentration–time profiles of silybin after oral administration of reference capsule (Enerthistle^®^) containing milk thistle raw material or HM40 capsule at a dose of 75 mg as silybin in healthy human subjects (*n* = 14).

Given the enhanced oral absorption of HM40 in rats, we examined the mean plasma concentration–time profiles of silybin after oral administration of reference capsule (Enerthistle^®^) or HM40 capsule at a dose of 75 mg as silybin in healthy human subjects (Figure 9). The mean pharmacokinetic parameters are listed in Table 3. The plasma concentration of silybin reached its maximum approximately 1 h after oral intake of the reference and HM40 capsules. Silybin was also rapidly absorbed in humans [63]; however, the absorption rate was slower than that in rats (T_max_: 0.25 h; Table 2), probably due to the delayed disintegration of the capsules. In addition, the MRT after the oral administration of the HM40 capsule was not significantly different from that of the reference capsule. These results could be due to the delayed disintegration and/or dissolution of silybin from the grapeseed oil soft capsules compared to the aqueous dispersion of HM40 in a rat study [64]. The values of C_max_ and AUC_inf_ after oral administration of the HM40 capsules were 2.05- and 1.51-fold higher, respectively, than those of the reference capsule, resulting in 1.5-fold higher oral bioavailability. 

This enhanced oral bioavailability in humans, consistent with the pattern observed in rats, could be attributed to the increased solubility and dissolution of silybin after preparation of the nanocrystal formulation. Therefore, we confirmed that the reduction in the particle size of milk thistle by the nanocrystal formulation (HM40) could facilitate its dispersion in the water phase and enhance its dissolution, thereby resulting in improved oral absorption of silybin in the GI tract.

## 4. Conclusions

A milk thistle nanocrystal formulation (HM40) composed of milk thistle raw material and excipients (xanthan gum and gum ghatti) at a 1:0.5 (*w*/*w*) ratio was prepared using a modified wet-milling method. It offered comparatively spherical and nano-sized particles when dispersed in water, without a change in the crystallinity of the API in the powder state. The surface morphology, crystallinity, mean particle size, silybin content, and silybin solubility of HM40 were maintained during the long-term storage for at least 24 months. The solubility of silybin and its dissolution from HM40 were significantly improved compared with those from the milk thistle raw material. In addition, the oral bioavailability of silybin from HM40 in rats and humans was enhanced by 2.61- and 1.51-fold, respectively, compared to that from the milk thistle raw material. Therefore, the milk thistle nanocrystal formulation could be useful for the oral delivery of poorly water-soluble silybin with enhanced solubility, dissolution, and bioavailability via the formation of nano-sized particles in the aqueous phase with good stability under long-term storage conditions.

## Figures and Tables

**Table 1:** The change of silybin content (% of initial day) from HM40, mean particle size, and polydispersity index (PDI) of HM40 dispersion during storage at 25 °C and 60% RH condition.

Time (Months)	Silybin Content (%, *w*/*w*)		Mean Particle Size (nm)	PDI
	% in Dried Powder	% of Initial Day		
0	26.0 ± 0.1	100	637 ± 43	0.464 ± 0.024
1	26.3 ± 0.5	101 ± 2	654 ± 39	0.486 ± 0.020
3	26.3 ± 1.3	101 ± 5	676 ± 9	0.495 ± 0.060
6	26.0 ± 0.3	100 ± 1	685 ± 35	0.433 ± 0.056
24	26.7 ± 0.5	103 ± 2	691 ± 43	0.283 ± 0.151

**Table 2:** Pharmacokinetic parameters of silybin after oral administration of milk thistle raw material or HM40 at a dose of 200 mg/kg as silybin in rats (*** *p* < 0.001, compared to milk thistle raw material).

Parameters	Milk Thistle Raw Material	HM40
T_max_ (h)	0.25	0.25
C_max_ (ng/mL)	703 ± 372	3320 ± 765 ***
AUC_inf_ (ng·h/mL)	1340 ± 488	3500 ± 617 ***
MRT (h)	3.74 ± 1.68	1.24 ± 0.41 ***
Relative bioavailability (%)	100	262

**Table 3:** Pharmacokinetic parameters of silybin after oral administration of reference capsule (Enerthistle^®^) containing milk thistle raw material or HM40 capsule at a dose of 75 mg as silybin in healthy human subjects (** *p* < 0.01; *** *p* < 0.001, compared to reference capsule).

Parameters	Reference Capsule	HM40 Capsule
T_max_ (h)	0.98 ± 0.50	1.03 ± 0.52
C_max_ (ng/mL)	158 ± 52	323 ± 121 **
AUC_inf_ (ng·h/mL)	209 ± 63	316 ± 72 ***
MRT (h)	1.91 ± 0.56	1.67 ± 0.58
Relative bioavailability (%)	100	151

## Data Availability

The data presented in this study are available on request from the corresponding author.

## Supplementary Materials

The following supporting information can be downloaded at: https://www.mdpi.com/article/10.3390/pharmaceutics16081033/s1, Figure S1: Mean particle size and size distribution of nanocrystal sheet (obtained after roller compressing and before milling) after dispersing in DI water.
