# Supplementary material for: Nanocrystal Formulation to Enhance Oral Absorption of Silybin: Preparation, In Vitro Evaluations, and Pharmacokinetic Evaluations in Rats and Healthy Human Subjects
Source: Pharmaceutics. 2024 Aug 2;16(8):1033. doi: 10.3390/pharmaceutics16081033 (PMC11359960; doi:10.3390/pharmaceutics16081033)
Supplement: Supplementary file 1 [file pharmaceutics-16-01033-s001.zip › pharmaceutics-3100849-supplementary.pdf]

Supplementary data

Supplementary Figure S1 Mean particle size and size distribution of nanocrystal sheet (obtained after roller compressing and before milling) after dispersing in DI water.

Abbreviations: DI, deionized water.

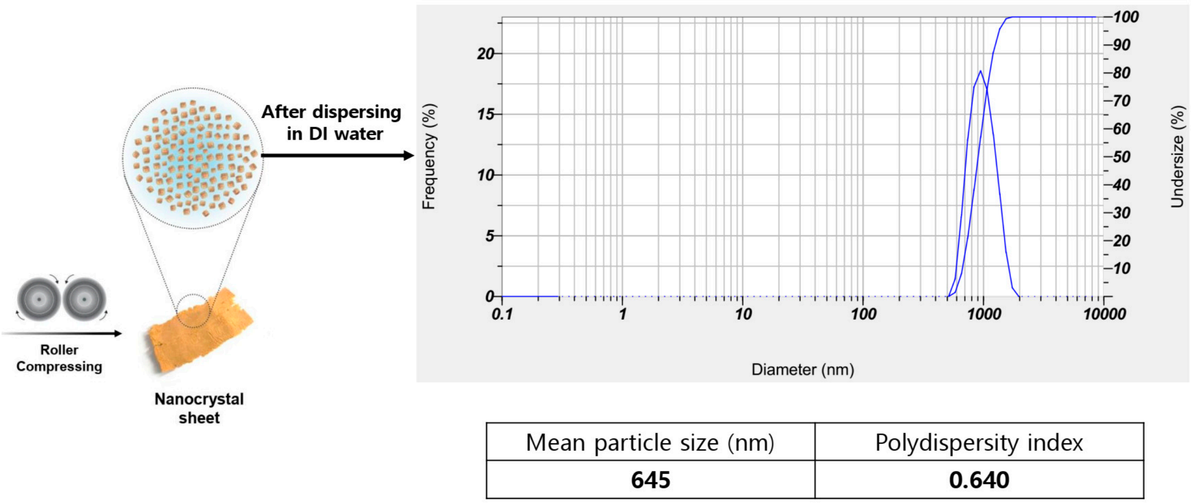

Mean particle size (nm)	Polydispersity index
**645**	**0.640**

Supplementary Figure S1
